# Does the timing of cardiac rehabilitation impact fitness outcomes? An observational analysis

**DOI:** 10.1136/openhrt-2015-000369

**Published:** 2016-08-01

**Authors:** Jennifer Fell, Veronica Dale, Patrick Doherty

**Affiliations:** Department of Health Sciences, University of York, York, UK

**Keywords:** QUALITY OF CARE AND OUTCOMES

## Abstract

**Objectives:**

To ascertain the characteristics associated with delayed cardiac rehabilitation (CR) and determine if an association between CR timing and fitness outcomes exists in patients receiving routine care.

**Methods:**

The study used data from the UK National Audit of Cardiac Rehabilitation, a data set which captures information on routine CR practice and patient outcomes. Data from 1 January 2012 to 8 September 2015 were included. Logistic regression models were used to explore the relationship between timing of CR and fitness-related outcomes as measured by patient-reported exercise level (150 min/week: yes/no), Dartmouth quality of life physical fitness scale and the incremental shuttle-walk test.

**Results:**

Based on UK data current CR practice shows that programmes do not always adhere to recommendations on the start of prompt CR, that is, start CR within 28 days of referral (42 days for coronary artery bypass graft (CABG)). Wait time exceeded recommendations in postmyocardial infarction (post-MI), elective percutaneous coronary intervention (PCI), MI-PCI and post-CABG surgery patients. This was particularly pronounced in the medically managed post-MI group, median wait time 40 days. Furthermore, statistical analysis revealed that delayed CR significantly impacts fitness outcomes. For every 1-day increase in CR wait time, patients were 1% less likely to improve across all fitness-related measures (p<0.05).

**Conclusions:**

With the potential for suboptimal patient outcome if starting CR is delayed, efforts should be made to identify and overcome barriers to timely CR provision.

**Key questions**

**What is already known about this subject?**

- Current guidelines state patients should be seen early, by the outpatient cardiac rehabilitation (CR) team, and start CR within 4 weeks of referral. Data show, however, that routine practice can deviate and delays occur, but the impact of this is unknown.

**What does this study add?**

- This multicentre analysis identified the characteristics of patients associated with delayed CR; notably, post-MI patients experience the longest delays. Analyses found that an association between timing of CR and patient response exists.

**How might this impact on clinical practice?**

- Given the importance of ‘exercise-based’ CR reducing mortality, it is important that programmes identify barriers and prioritise timely pathways of care to prevent avoidable delays to the start of CR.

## Introduction

Cardiovascular diseases are common and burdensome, responsible for an estimated 30% (17.5 million) of all deaths globally in 2012 and costing an estimated £18.9 billion in the UK during 2014.^1^ ^2^ Based on national and international guidelines, cardiac rehabilitation (CR) is offered as an effective secondary prevention intervention, proven to reduce premature cardiovascular and all-cause mortality and improve health-related quality of life (QoL).^3–5^ CR is also a cost-effective therapy with an estimated cost per life year gained of less than £2000.^6^

The National Audit of Cardiac Rehabilitation (NACR), funded by the British Heart Foundation, is a database which facilitates the monitoring of CR services in the UK in terms of service delivery and patient outcome. In 2014, 311 programmes were identified as delivering a core CR programme and 257 provided data to the NACR.^7^ Despite clinical minimum standards published in the UK and Europe, variation in practice can be observed, including the timing of CR.^5^ ^7^ ^8^ Deviation from evidence-based standards may be accounted for by increasing demands on programmes and decreasing resources.^9^ There is, however, a perception that such delays may not only reduce the chances of enrolment but also the impact of CR, and emerging evidence appears to demonstrate this may be the case.^10–12^

Current guidance states patients should be seen early, by the outpatient CR team, and start CR within 4 weeks of referral.^3^ ^5^ ^8^ ^9^ ^13^ Timing deviations occur in practice, but to date, it is unclear what the impact of such digressions from clinical guidelines could be. This study will ascertain the characteristics associated with delayed CR and the association between CR timing and patient outcome, namely physical activity status and fitness outcomes. Physical activity-related outcomes are especially critical given the emphasis of exercise-based CR reducing mortality.^4^ Findings from this project will establish if prioritisation of wait time reductions should take precedence.

## Methods

This observational study is reported following the guidelines: Strengthening the Reporting of Observational Studies in Epidemiology (STROBE).^14^ In the UK, CR is delivered in accordance with national standards, running for a minimum of 8 weeks or 56 days (median duration of CR 51–56 days^7^) and comprising of a multidisciplinary team based either in the community or an outpatient hospital setting.^8^ ^13^ The aim of CR is to facilitate health behaviour change through supervised exercise, educational classes on risk factors, physical activity, diet and smoking cessation and psychosocial support.

As part of routine practice, programmes undertake baseline and post-CR assessments, shortly after CR completion, to monitor progress in patients. Centres across England, Wales and Northern Ireland enter data into NACR, varying in size and case mix providing a representative sample. Data are collected and hosted by the Health and Social Care information Centre. Through annual data sharing agreements, approval is granted to use these data to monitor and report on the quality of CR. Analyses were conducted using all available data from centres across the UK, to minimise selection bias, which entered data into NACR from 1 January 2012 to 8 September 2015.

### Participants

**Figure 1:**
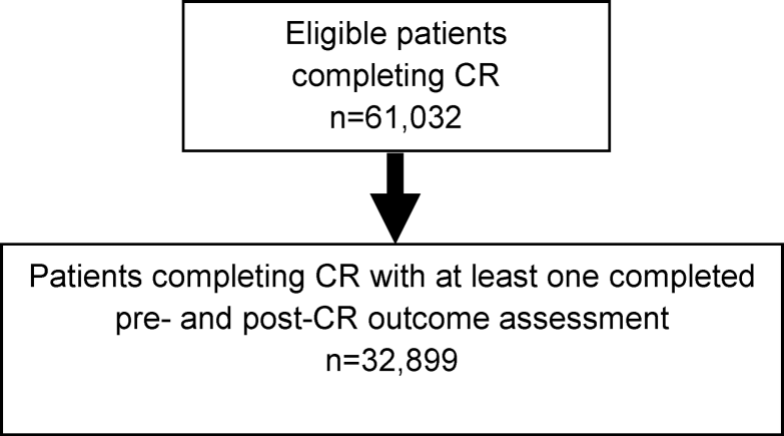
Patient flow diagram. CR, cardiac rehabilitation.

Figure 1 details the flow of patients in this study. Adult (≥18 years) patients with acute coronary syndrome (ACS) starting CR from one of four patient groups were included: medically managed postmyocardial infarction (post-MI), elective percutaneous coronary intervention (PCI), MI-PCI and postcoronary artery bypass graft (CABG) surgery. Patients were defined as completing CR if the duration of CR exceeded 7 days and a completion date was entered. Only patients starting CR, attending a pre-CR and post-CR assessment with at least one completed physical activity outcome measure were included.

### Timing categories

CR timing (ie, time between referral and start of core CR) was included in the analyses as a continuous variable to determine the impact on outcome for every day increase in CR wait time. A separate analysis investigated the impact of CR timing according to the definition of ‘early’ or ‘late’ CR as per current recommendations, that is, start within 4 weeks of referral. For this, a categorical CR timing variable was generated as follows:

- CR ‘on time’ (0–28 days),
- Delayed CR (29–365 days).

Timing was adjusted for CABG patients, where recovery from surgery (eg, sternotomy) is an important step before rehabilitation can start. Timing groups for CABG patients were as follows: on time (0–42 days) and delayed CR (43–365 days).

### Outcome measures

Patient-reported physical activity level (150 min/week: yes/no), Dartmouth Quality of Life in relation to physical fitness (healthy status score 1–3/non-healthy status score 4–5)^15^ and a direct measure of fitness the incremental shuttle-walk test (ISWT), which assesses how far and fast a patient can walk without stopping while walking speed is gradually increased,^16^ were included. These outcomes capture both a patient-reported perspective and a clinician-assessed measure of fitness. Change in distance (metres) before CR and after completion of CR was calculated for the ISWT and categorised into <70 m improvement in distance or ≥70 m distance improvement. This cut-off is based on a recent study which proposes a 70 m improvement in distance as the minimum considered meaningful to a patient.^16^

### Statistical analysis

All analyses were conducted using STATA V.13.1. Descriptive statistics were generated for early and late CR groups and compared for statistical significant differences using χ^2^ test, student t test or Wilcoxon rank-sum test as appropriate. Logistic regression was performed to investigate the relationship between CR timing and patient outcome after CR completion. Analyses accounted for known confounders of fitness: age, gender, number of comorbidities, duration of CR (days), baseline body mass index (BMI), systolic and diastolic blood pressure (mm Hg), smoking status (smoker/non-smoker), ethnicity (British, non-British), treatment (revascularised or medically managed) and baseline physical activity level. To take account of the nested nature of the data, that is, patients treated within centres, the Huber-White-sandwich estimator robust SEs method was used.

## Results

Patient characteristics are presented in table 1. As typical in the UK,^7^ CR was accessed primarily by older British males with at least one comorbidity. Physical activity level was generally low at baseline with only 33% of patients reporting at least 150 min of physical activity per week. The median duration of CR received was 57 days, which meets the minimum standard of 8 weeks (56 days).^8^ ^13^

**Table 1:** Patient characteristics at baseline (pre-CR) overall and by early and late CR groups

Baseline characteristic	Overall (n=32 899)	Early CR (n=12 254)	Late CR (n=20 645)
Mean age, years (SD)	64.91 (10.73)	63.86 (10.76)	65.54 (10.67)*
Gender, n males (%)	25 012 (77)	9467 (79)	15 545 (76)**
Ethnicity, n British (%)	23 191 (86)	8792 (87)	14 399 (85)*
Post-MI (%)	4280 (13)	1313 (11)	2967 (14)**
MI-PCI (%)	13 331 (40)	4774 (39)	8557 (41)**
PCI (%)	7505 (23)	3320 (27)	4185 (20)**
CABG (%)	7783 (24)	2847 (23)	4936 (25)
Mean body mass index (SD)	27.99 (4.73)	28.09 (4.73)	27.93 (4.74)*
One or more comorbidities, n (%)	23 527 (72)	8469 (69)	15 058 (73)**
Mean systolic blood pressure (SD)	129.34 (20.07)	128.60 (19.58)	129.78 (20.34)*
Mean diastolic blood pressure (SD)	74.31 (11.37)	74.53 (11.15)	74.19 (11.50)*
Non smoker, n (%)	18 010 (89)	7354 (90)	10 656 (88)**
Physical activity ≥150 min/week, n (%)	9976 (33)	3976 (35)	6000 (32)**
Healthy fitness status on QoL, n (%)	11 373 (44)	4237 (42)	7136 (45)**
Median shuttle-walk distance	350 m	360 m	340 m**

*p≤0.005 versus early CR.

**p≤0.001 versus early CR.

CABG, coronary artery bypass graft; CR, cardiac rehabilitation; MI, myocardial infarction; PCI, percutaneous coronary intervention; QoL, quality of life.

Patients starting CR late were statistically significantly more likely to be older, female, non-British, lower BMI, at least one comorbidity, higher systolic blood pressure, lower diastolic blood pressure, currently smoke, low physical activity level (<150 min/week) and shorter baseline ISWT distance (all p≤0.05). Participants in both early and late CR groups were predominantly patients with MI-PCI. In terms of patient improvement following CR, the extent of benefit was smaller for late CR attenders across the three fitness-related outcomes. In early CR attenders, the proportion achieving healthy physical activity levels and normal fitness-related QoL improved by 31% and 36%, respectively. Median improvement in ISWT was 120 m. For late CR, attenders values were 27%, 29% and 90 m, respectively.

**Figure 2:**
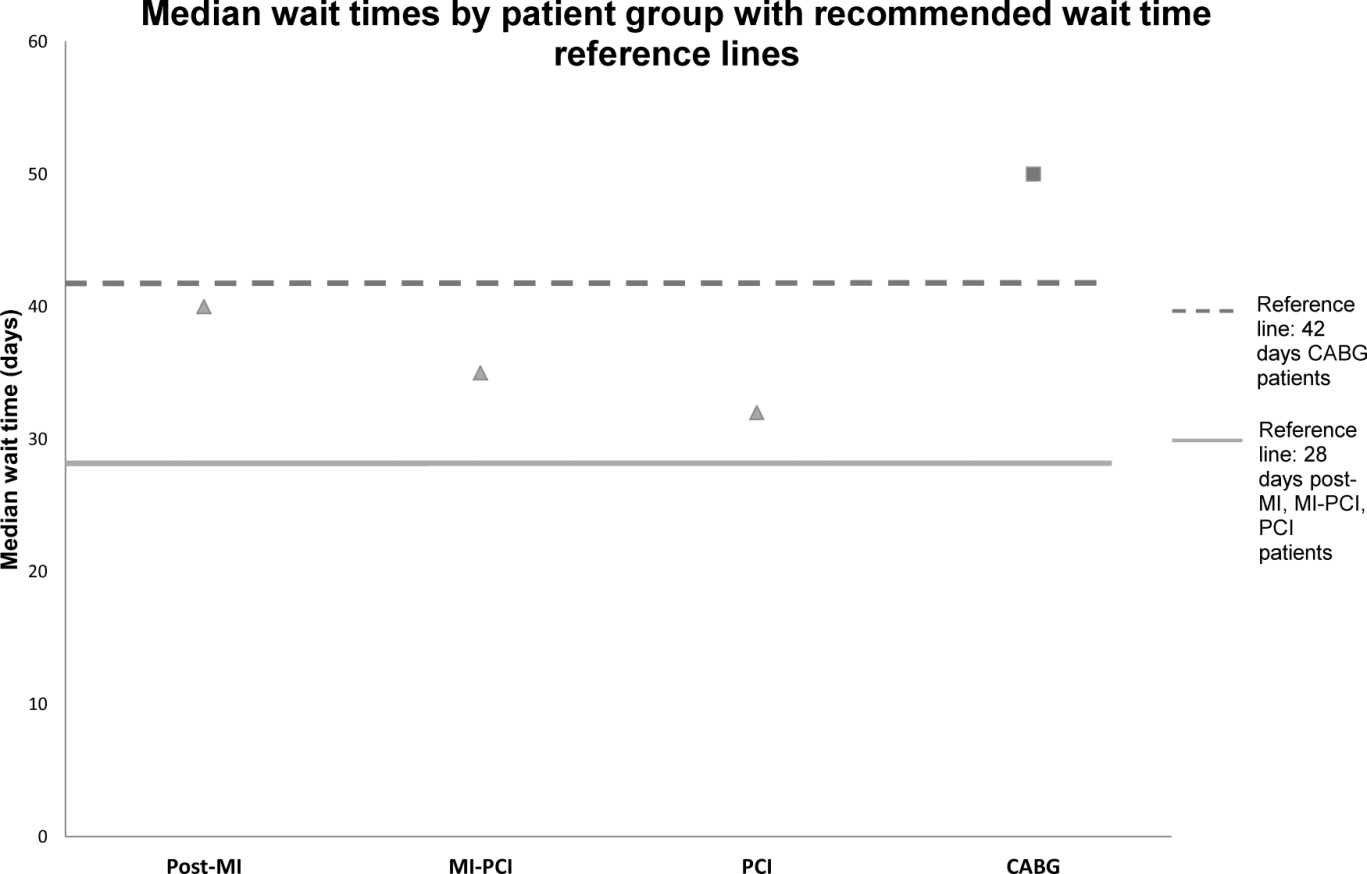
Median wait times by patient group with recommended wait time reference lines. CABG, coronary artery bypass graft; MI, myocardial infarction; PCI, percutaneous coronary intervention.

The median wait time between CR referral and CR start exceeded recommendations at 39 days, with 63% of the population classified as late starters of CR. The proportion of delayed patients was 69% for MI, 64% MI-PCI, 56% for PCI and 63% for CABG patients. Figure 2 presents the median wait time (days) by patient group against recommended wait times (28 or 42 days for CABG). In each patient group, median wait time exceeded the recommended maximum waiting time, the delay was particularly extended in the post-MI population, which exceeded the maximum recommended wait time by 12 days.

### Outcomes

The findings from the logistic regression analyses are presented in table 2. After multivariate adjustment, late CR timing was found to be a significant independent predictor of decreased fitness level compared with early CR. Similarly, CR timing when included as a continuous measure was also a significant predictor.

**Table 2:** Results from logistic regression—relationship between CR timing and patient outcome post-CR

	OR	Significance	95% CI
CR timing (days)			
Physical activity status (150 min)	0.997	0.005	0.995 to 0.999
Physical fitness QoL	0.996	<0.001	0.995 to 0.998
Shuttle-walk test	0.997	0.003	0.995 to 0.999
Late CR			
Physical activity status (150 min)	0.863	0.051	0.744 to 1.000
Physical fitness QoL	0.773	0.001	0.668 to 0.893
Shuttle-walk test	0.793	0.008	0.669 to 0.941

Analyses adjusted for age, gender, number of comorbidities, duration of CR (days), BMI, systolic and diastolic blood pressure, smoking status, ethnicity, treatment, baseline fitness status.

OR, p value and 95% confidence intervals (CI).

BMI, body mass index; CR, cardiac rehabilitation; QoL, quality of life.

## Discussion

Current guidelines and papers in cardiac care recommend the early start of CR where appropriate.^3^ ^5^ ^8–10^ ^13^ ^17^ However, evidence shows in some cases, there is disconnect between recommended practice and the ‘real-life’ conduct of CR. Overall, 63% of our study population were classified as late CR attenders and median wait times in each patient group exceeded maximum recommendations on wait time. One explanation for the higher proportion of late attenders may be comorbidity burden. A total of 73% of late CR attenders had at least one comorbidity compared with 69% in early attenders. Case complexity could certainly delay the start of rehabilitation. Inconsistency in delay time across groups is also concerning. Out of all the patient groups, post-MI patients notably exceeded the maximum recommended wait time by the largest number of days (12 days). This may seem contrary to expectations, as post-MI patients undergo no invasive revascularisation procedures, which can delay CR start due to recovery period.

Regardless of CR timing, improvements in fitness-related outcomes were observed pre-CR to post-CR. However, the extent of improvement was reduced in late CR attenders. To explore the impact of CR timing on outcome in detail, two analytical approaches were used; timing as a continuous measure to explore the relationship between increasing wait time and outcome and timing as a categorical measure (early/late) to explore the relationship in the context of current guidelines. In each approach, it was observed that CR timing was a significant predictor of patient outcome in terms of fitness level. Based on these analyses, the likelihood of reporting a positive physical activity level and fitness outcome was reduced when CR is delayed. This was consistent regardless of whether the measure was patient reported or clinician assessed. This seems to fit with a recent study of 1241 CR patients which concluded delayed enrolment is directly related to patient outcome (metabolic equivalent of tasks (METs) and weight improvement).^10^ Additional evidence has also suggested that lifestyle changes peak in the first 6 months for patients with ACS patients undergoing exercise and lifestyle interventions, thus timing of CR is critical to optimise response.^18^

Furthermore, a number of studies have shown several outcomes can be positively influenced by starting CR early, including mortality and cardiovascular events reductions,^19^ functional improvements, cardiorespiratory measures, 6 min walk test, QoL^20–22^ and cardiac functioning,^23–25^ with each outcome showing a greater improvement from early CR practice. The safety of early enrolment has also recently been assessed in open heart patients finding no difference in major event rates between early and late enrolees to CR.^17^ Clearly the case for early CR is strong, perhaps even to the point that a reduction in the recommended wait times may be warranted. Aside from clinical outcomes, additional evidence suggests that CR timing may even impact initial enrolment to CR. One randomised controlled trial found an early CR orientation session increased attendance by 18%. A further investigation into wait time and enrolment, using routine patient records, also reported an association; for every 1-day increase in wait time patients were 1% less likely to enrol.^11^ ^12^

Given the potential implications to CR attendance and the importance of delivering a successful CR ‘exercise component’, any factors which negatively influence the extent of success of fitness-related outcomes, such as a delayed CR start, should be avoided if possible.

### Limitations

To our knowledge, this is the first large-scale, multicentre analysis (n=32 899 eligible patients) which has investigated the effects of delayed CR timing on patient outcomes using routinely collected UK patient data. Although CR programmes are encouraged to provide complete patients records, it was expected that a proportion of patient data would be missing due to non-completion of patient records. The demographics of those included in the analyses were, however, typical of the UK population accessing CR.^7^ In addition, analyses were adjusted for a number of confounding measures which may influence physical activity status and fitness outcomes. Disease severity was not included, as this is not captured in the NACR database; however, comorbidities and other baseline characteristics will have partially accounted for this.

## Conclusion

The observed association of CR timing and patient outcome in these analyses provides evidence to support the continued need for timely CR as directed by current guidance. The annual NACR report shows many programmes are not delivering timely CR and in these instances barriers need to be identified and overcome to ensure a consistent and effective service. Notably post-MI patients appear to experience the greatest delays and this should be investigated further. The clear association between exercise-based CR and reduced mortality means it is especially important that any potential causes of suboptimal improvement in fitness are avoided.^4^ Although it is acknowledged that timing of CR should also be based on a case-by-case basis, care should be taken to prevent avoidable delays, that is, long waiting lists. Future research should also consider the effects of mode of CR delivery on patient outcomes.

## References

[1] World Health Organisation. Cardiovascular diseases factsheet 317. WHO, 2015.

[2] Centre for Economics and Business Research. The economic cost of cardiovascular disease from 2014–2020 in six European economies. London: CEBR, 2014.

[3] NICE. MI-secondary prevention guideline 172. UK: NICE, 2013.

[4] Heran BS, Chen JM, Ebrahim S Exercise-based cardiac rehabilitation for coronary heart disease. Cochrane Database Syst Rev 2011;(7):CD001800. 21735386 10.1002/14651858.CD001800.pub2 PMC4229995

[5] Piepoli MF, Corrà U, Adamopoulos S Secondary prevention in the clinical management of patients with cardiovascular diseases. Core components, standards and outcome measures for referral and delivery: a Policy Statement from the Cardiac Rehabilitation Section of the European Association for Cardiovascular Prevention & Rehabilitation. Endorsed by the Committee for Practice Guidelines of the European Society of Cardiology. Eur J Prev Cardiol 2014;21:664–81. 10.1177/2047487312449597 22718797

[6] Fidan D, Unal B, Critchley J Economic analysis of treatments reducing coronary heart disease mortality in England and Wales, 2000–2010. QJM 2007;100:277–89. 10.1093/qjmed/hcm020 17449875

[7] The National Audit of Cardiac Rehabilitation (NACR). Annual Statistical Report. UK: NACR, 2014.

[8] BACPR. The BACPR standards and core components for cardiovascular disease prevention and rehabilitation. UK: BACPR, 2012. 10.1136/heartjnl-2012-303460 23403407

[9] Department of Health. Service specification for cardiac rehabilitation services. UK: DoH, 2010.

[10] Johnson DA, Sacrinty MT, Gomadam PS Effect of early enrollment on outcomes in cardiac rehabilitation. Am J Cardiol 2014;114:1908–11. 10.1016/j.amjcard.2014.09.036 25438920

[11] Pack QR, Mansour M, Barboza JS An early appointment to outpatient cardiac rehabilitation at hospital discharge improves attendance at orientation: a randomized, single-blind, controlled trial. Circulation 2013;127:349–55. 10.1161/CIRCULATIONAHA.112.121996 23250992

[12] Russell KL, Holloway TM, Brum M Cardiac rehabilitation wait times: effect on enrollment. J Cardiopulm Rehabil Prev 2011;31:373–7. 10.1097/HCR.0b013e318228a32f 21826016

[13] NICE. Cardiac rehabilitation services commissioning guide 40. UK: NICE, 2011.

[14] von Elm E, Altman DG, Egger M, STROBE Initiative. Strengthening the Reporting of Observational Studies in Epidemiology (STROBE) statement: guidelines for reporting observational studies. BMJ 2007;335:806–8. 10.1136/bmj.39335.541782.AD 17947786 PMC2034723

[15] Wasson J, Keller A, Rubenstein L Benefits and obstacles of health status assessment in ambulatory settings. The clinician's point of view. The Dartmouth Primary Care COOP Project. Med Care 1992;30(5 Suppl):Ms42–9. 1583940 10.1097/00005650-199205001-00004

[16] Houchen-Wolloff L, Boyce S, Singh S The minimum clinically important improvement in the incremental shuttle walk test following cardiac rehabilitation. Eur J Prev Cardiol 2015;22:972–8. 10.1177/2047487314540840 24958737

[17] Pack QR, Dudycha KJ, Roschen KP Safety of early enrollment into outpatient cardiac rehabilitation after open heart surgery. Am J Cardiol 2015;115:548–52. 10.1016/j.amjcard.2014.11.040 25543236

[18] Chow CK, Jolly S, Rao-Melacini P Association of diet, exercise, and smoking modification with risk of early cardiovascular events after acute coronary syndromes. Circulation 2010;121:750–8. 10.1161/CIRCULATIONAHA.109.891523 20124123

[19] Rauch B, Riemer T, Schwaab B Short-term comprehensive cardiac rehabilitation after AMI is associated with reduced 1-year mortality: results from the OMEGA study. Eur J Prev Cardiol 2014;21:1060–9. 10.1177/2047487313486040 23559535

[20] Eder B, Hofmann P, von Duvillard SP Early 4-week cardiac rehabilitation exercise training in elderly patients after heart surgery. J Cardiopulm Rehabil Prev 2010;30:85–92. 10.1097/HCR.0b013e3181be7e32 19952770

[21] Teasell R, Bitensky J, Salter K The role of timing and intensity of rehabilitation therapies. Top Stroke Rehabil 2005;12:46–57. 10.1310/ETDP-6DR4-D617-VMVF 16110427

[22] Stoller O, de Bruin ED, Knols RH Effects of cardiovascular exercise early after stroke: systematic review and meta-analysis. BMC Neurol 2012;12:45 10.1186/1471-2377-12-45 22727172 PMC3495034

[23] Giallauria F, Acampa W, Ricci F Effects of exercise training started within 2 weeks after acute myocardial infarction on myocardial perfusion and left ventricular function: a gated SPECT imaging study. Eur J Prev Cardiol 2012;19:1410–19. 10.1177/1741826711425427 21965517

[24] Giallauria F, Acampa W, Ricci F Exercise training early after acute myocardial infarction reduces stress-induced hypoperfusion and improves left ventricular function. Eur J Nucl Med Mol Imaging 2013;40:315–24. 10.1007/s00259-012-2302-x 23224706

[25] Haykowsky M, Scott J, Esch B A meta-analysis of the effects of exercise training on left ventricular remodeling following myocardial infarction: start early and go longer for greatest exercise benefits on remodeling. Trials 2011;12:92 10.1186/1745-6215-12-92 21463531 PMC3083361

